# Gamma-Radiation-Induced Endoplasmic Reticulum Stress and Downregulation of WFS1, Nectin 3, and Sostdc1 Gene Expression in Mice Hippocampus

**DOI:** 10.32598/bcn.9.10.205

**Published:** 2019-07-01

**Authors:** Jaldeep Langhnoja, Mohammed Mustak

**Affiliations:** 1. Department of Applied Zoology, Faculty of Science & Technology, Mangalore University, Mangalagangothri, Karnataka State, India.

**Keywords:** Gamma radiation, Hippocampus, Endoplasmic reticulum stress, Nectin 3

## Abstract

**Introduction:**

Neurogenesis mainly occurs in the hippocampus that is sensitive to radiation. More histological changes are reported at higher doses of radiation, while low dose radiation causes cognitive dysfunction in adult mammals. In the present study, we tried to correlate the Endoplasmic Reticulum (ER) stress-mediated hippocampus dysfunction after whole-body gamma radiation of mice.

**Methods:**

Mice were exposed to a series of gamma radiations, followed by isolation of hippocampus. To elucidate the gene expression profile, qPCR was performed for ER stress markers CHOP, BiP, and hippocampal specific genes WFS1, Nectin 3, and Sostdc 1 on the isolated hippocampus. Expression of CHOP and ERK½ were analyzed by western blot on exposure to gamma radiation.

**Results:**

qPCR results showed a significant increase in the expression of ER stress-specific genes CHOP, BiP, and decrease in hippocampal specific genes WFS1, Nectin3, and Sostdc1. Western blot study suggests a significant increase in ER stress proteins like CHOP and ERK½ expression.

**Conclusion:**

Exposure to gamma radiation significantly increased the expression of ER-stress genes, suggesting that ER stress plays a major role in inducing radiation mediated dysfunction of the hippocampus. Also, significant downregulation of WFS1, Nectin3, and Sostdc1 genes suggests radiation mediated effect of hippocampal CA 1, CA 2, and CA 3 regions. A further significant increase of ERK½ shows involvement of the ERK pathway in mediating radiation-induced ER stress dysfunction in mice hippocampus. The present findings may lead to the identification of ER stress as a new marker to study radiation-induced neurodegenerative disorder.

## Highlights

- Gamma-irradiation exposure causes significant endoplasmic reticulum (ER) stress-mediated apoptosis in mice hippocampus.
- WFS1 gene plays a part in inducing ER stress-mediated dysfunction in the hippocampus.
- Downregulation of nectin3 also suggests hampered episodic memories and loss of neurogenesis in the CA3 region of the hippocampus.
- Sostdc1 gene, which is predominantly found in the CA2 region of the hippocampus, is also reported to play a major role in the Wnt receptor signaling pathway.
- Upregulation of ERK ½ protein expression post 10-Gy exposure of gamma radiation suggests ERK pathway-mediated cell death, which might be due to phosphorylation of pro-apoptotic signal.

## Plain Language Summary

Myelination is essential for normal brain functions. The significance of various radiation-induced brain disorders has been more apparent recently because of the pathology of numerous disorders of the brain in people exposed to various kind of ionizing radiation directly or indirectly. Furthermore, patients who have schizophrenia show a decreased density of glial cells and memory loss. The endoplasmic reticulum stress response is mediated by chaperone proteins and cause an imbalance in brain homeostasis. This study aims to understand the influence of Gamma-irradiation on mouse brain and study how molecular markers can be targeted for the early identification of radiation-induced brain disorders.

## 1. Introduction

In various clinical conditions, the brain gets exposed to ionizing radiation. Though radiotherapy is considered as one of the primary treatment modality in different pathological conditions, the potential injury to normal tissue is unavoidable. Radiation exposure often causes a wide array of cognitive dysfunctions in adult and pediatric tumor patients (Merchant, Pollack, & Loeffler, 2010). Ionizing Radiation (IR) has a wide plethora of effects in both young and adult brain. More histological changes are reported at higher doses of radiation, while low dose radiation causes cognitive dysfunctions in adult mammals (Raber et al., 2004). Repeated exposure to various stresses leads to the adverse effect in cognition across multiple life stages (Lupien, McEwen, Gunnar, & Heim, 2009).

Hippocampus is considered to be one of the major sites for active neurogenesis (Van Praag, 2005; Mizumatsu et al., 2003). Hippocampal CA1, CA2, CA3 areas play a significant role in maintaining CNS homeostasis and are involved in various physiological processes (Frederick Hitti, Siegelbaum, 2014; Jensen & Lisman, 1996; Dudek, Alexander, & Farris, 2016; Meyer et al., 2014). Nectin-3 an immunoglobulin-like cell adhesion molecule which is mainly localized in the CA3 pyramidal neurons plays an important role in the synaptic formation, maintenance, and remodeling (Mizoguchi et al., 2002; Honda et al., 2006; Thompson et al., 2008).

Studies indicate that exposure to ionizing radiation could induce ultrastructural modifications in the ER (Boraks, Tampelini, Pereira, Chopard, 2008). ER forms the major protein folding machinery in the cell. Cell homeostasis gets disrupted when the load of the unfolded protein increases and Unfolded Protein Response (UPR) pathway fails to repair the misfolded protein which leads to the accumulation of these proteins in the ER lumen (Taniguchi, Yoshida. 2015). Accumulation of these misfolded proteins has been proved to cause apoptosis (Oakes, Papa. 2015), which ultimately leads to neurodegenerative diseases. The UPR pathway usually is active as self-defense machinery in the cell, which increases the secretion of molecular chaperones such as BiP and GRP78, which belongs to the heat shock protein family and foldases. However, when misfolded proteins accumulate in the excessive amount, they may overwhelm the quality control machinery.

The mammalian UPR directs the cell to an apoptotic pathway, leading to cell death. C/EBP Homologous Protein (CHOP), also known as GADD153 (growth arrest- and DNA damage-inducible gene 153), is triggered by ER stress. CHOP overexpression triggers cell cycle arrest and apoptosis, down-regulates the pro-survival molecule Bcl-2, and promotes the production of reactive oxygen species (Marciniak et al., 2004). Quite the opposite, overexpression of the ER chaperone BiP reduces CHOP induction that is associated with ER stress and attenuates apoptosis (Wang et al., 2013).

Another player, WFS1, is a transmembrane protein present in the ER is shown to play a significant role in mitigating ER stress response in the cell (Takeda et al., 2001). Wolfram syndrome a genetic condition of diabetes, optic atrophy neurodegeneration, and psychiatric illness, is reported to be caused by mutation of WFS1 gene (Strom et al., 1998; Inoue et al., 1998). Reports also suggest that the increased level of ER-stress signaling leads to cell death, causing neuronal dysfunction in the Wolfram syndrome (Yamada et al., 2006; Riggs et al., 2005; Kakiuchi, Ishiwata, Hayashi, & Kato, 2006).

Sclerostin Domain Containing 1 (Sostdc1) belongs to a Bone Morphogenetic Protein (BMP) antagonist. In the development of cultured sympathetic and cerebellar neurons, the BMP family of proteins play a significant role by inducing synaptogenesis and dendritic growth (Lein et al., 2002). An altered level of Sostdc1 contributes to various disease conditions (Park et al., 2009). Sostdc1 gene has also been reported in thapsigargin-induced ER stress in mouse osteoblasts (Hamamura, Liu, & Yokota, 2008). However, the underlying mechanism is still unclear.

Cell Adhesion Molecules (CAM) are the principal constituent of synapses and also the modulators of synaptic activity and plasticity (Shapiro, Love, & Colman, 2007). Nectin-3 is a class of immunoglobulin-like CAM that presents in both postsynaptic and presynaptic and is connected to the actin cytoskeleton via L-afadin. The nectin-afadin complex coordinates with the cadherin-catenin junction and participates in synaptic formation, remodeling, and maintenance (Mizoguchi et al., 2002; Honda et al., 2006). Evidence also suggests impaired nectin-mediated damage in hippocampal development and mental retardation (Park et al., 2009). Nectin-3 is abundantly present in CA3 region of the hippocampus (Thompson et al., 2008) and is vulnerable to acute and chronic stress (Mizoguchi et al., 2002; Suzuki et al., 2000). Protein kinases play a crucial role in various signaling networks to maintain cell homeostasis and its functions.

Mitogen-Activated Protein Kinase (MAPK) has a conserved function and contributes to various hippocampus-mediated neurodegenerative diseases (Giovannini et al., 2008). Extracellular-signal-Regulated kinase½ (ERK½) is one of the members of the MAPK family and has been spotted in various disease conditions. Ultraviolet irradiation activates ERK½ in various primary immortalized and transformed cells (Tang et al., 2002). However, radiation-induced changes in hippocampal Nectrin3, WFS1, and Sostdc1 gene expression in ER stress condition via ERK½ pathway are still unclear. To the best of our knowledge, there is no report on how radiation induces ER stress-mediated alteration in the hippocampus of mice exposed to whole-body radiation. In the present study, we have tried to understand the ER stress-mediated changes in mice hippocampus after exposing to whole-body gamma radiation.

## 2. Methods

### 2.1 Study subjects

Adult Swiss albino mice (Mus musculus) were housed in pairs under standard laboratory conditions with artificial 12 h light/dark cycle at an ambient temperature of 25°C–27°C with free access to food and water. All experiments were conducted following the ethical guidelines by the Committee for the Purpose of Control and Supervision of Experiments on Animals, Government of India and cleared by the Institutional Animal Ethics Committee.

### 2.2. Gamma irradiation

For irradiating the samples, 60Co-gamma chamber-1200 supplied by Board of Radiation and Isotope Technology (BRIT), DAE, Mumbai was used in the Centre for Application of Radiation and Radioisotope Technology (CAART, Mangalore University). The dose rate of the above the gamma chamber was measured and found 10.2333 Gy/min using Fricke dosimetry system (Nairy, Bhat, Sanjeev, & Yerol, 2016). For the experiment, 6–8 weeks old matched (weighing: 25±2 g) male Swiss albino mice (Mus musculus) were used. All animals were supplied with standard mice food and water ad libitum.

Jagetia et al. (2003) reported, 6- to 12-Gy intensity of gamma radiation significantly increase lipid peroxidation and depletion of Glutathione (GSH) in mice exposed to whole-body radiation. In the present study, the mice were exposed to gamma radiation in dose ranges of 7 Gy, 8 Gy, 9 Gy, 10 Gy in a well ventilated restrained perplex box. After exposure, the animals were kept for 24 h and then sacrificed and their hippocampus were isolated for further analysis. The above experimental protocol was approved by the Institutional Animal Ethics Committee (IAEC) of Mangalore University.

### 2.3. Isolation of hippocampus

After killing the mice, their brains were immediately dissected out on the ice and placed in a pre-chilled stereotaxic brain block (Kopf, USA). One-millimeter thick sections of the hippocampus were serially cut out using Paxinos and Watson atlas. The parts of some sections of the hippocampus are stored in RNA Later Solution (Invitrogen) for gene expression studies and RIPA buffer (Himedia) for protein expression studies. For both Real-Time qPCR and western blot analysis, 5 adult Swiss albino mice were kept in each experimental group.

### 2.4. Real-time qPCR analysis

Total RNA was isolated from cells by TRIzol reagent (Invitrogen). The Qubit RNA assay kit (Invitrogen) was used for quantifying the isolated RNA. One microgram of the total RNA was used for a 20 μL reaction. The Verso cDNA synthesis kit (Applied Biosystems) was used for the Reverse Transcription (RT) reaction. For the quantitative RT-PCR, SYBR Select Master Mix (Applied Biosystems) was used in QuantStudio 12K (Life Technology) real-time PCR machine with primers specific to detect the target messenger RNA (mRNA).

### 2.5. Western blot

The western Blot analysis was carried out to understand the expression level of CHOP and ERK pathway on exposure to IR in mice hippocampus. The tissues were lysed using lysis buffer (Himedia) and stored at −20°C for further analysis. Qubit protein assay kit (Invitrogen) in Qubit 2.0 fluorometer (Invitrogen) was used to quantify the isolated protein from the hippocampus tissue homogenate. About 40 μg of the quantified protein sample was dissolved in 10% SDS polyacrylamide gel and further transferred to the nitrocellulose membrane. After transfer, the membrane was blocked using 3% BSA in Tris-buffered saline and Tween 20 mixture (0.2 %) and incubated in primary antibody overnight at 4°C. The primary antibodies of anti-GAPDH (1:1000, Abchem), anti-CHOP (1:1000, Cell signaling), and anti-ERK½ (1:1000, Pierce) were used. The bands were visualized in ChemiDoc (Bio-Rad) using corresponding horseradish peroxidase-conjugated secondary antibodies (Sigma). The bands were quantified using ImageJ software and graphs were plotted by GraphPad Prism-3 software.

### 2.6. Statistical analysis

Statistical analysis was performed by 1-way ANOVA followed by Dunnett’s multiple range test in Prism 3 software (GraphPad Software Inc.). The data were expressed as Mean±SD. P values less than 0.05 were considered statistically significant (*P<0.05; **P<0.01; ***P<0.001).

## 3. Results

### 3.1. Effect of radiation on ER stress and hippocampus specific gene expression

**Figure 1.**
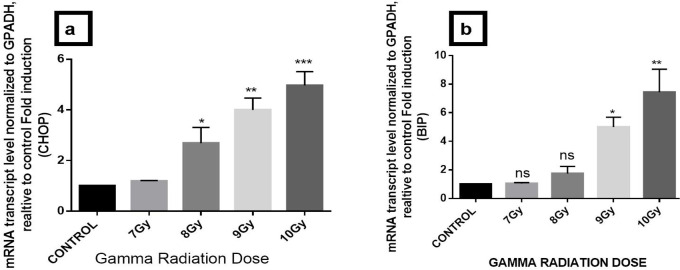
qRT-PCR analysis of isolated hippocampus on exposure to whole-body gamma radiation of mice. The qRT- PCR analysis of the transcript levels of ER stress-specific gene a. CHOP; and b. BiP on exposure to 7 Gy, 8 Gy, 9 Gy, and 10 Gy radiation. GAPDH was used as an internal control for the estimation of target gene expression. For quantitative representation, the graph is plotted as gene expression compared to the control. Then, 1-way ANOVA, followed by Dunnett’s multiple, were used to check the significance based on the control. Error bars represent Mean±SD with ANOVA parameters (^*^P<0.05; ^**^P<0.01; ^***^P<0.001).

**Figure 2.**
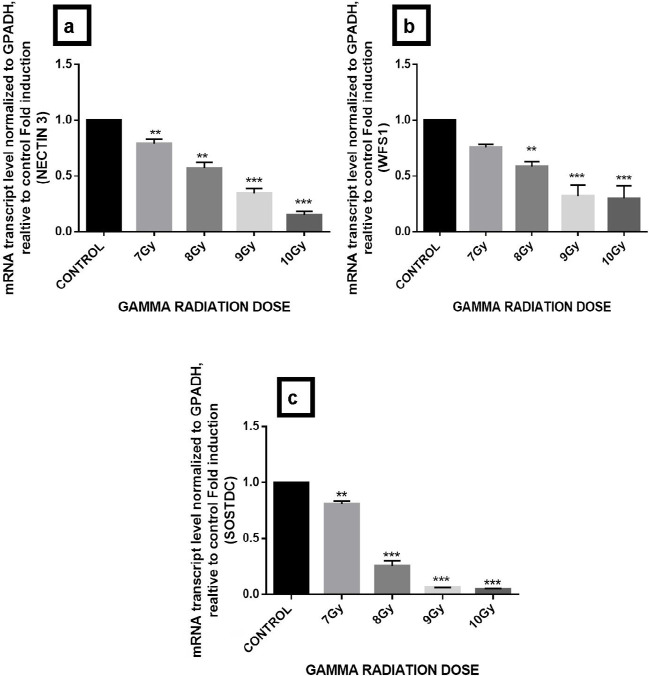
qRT-PCR analysis of the transcript levels of a. Nectin3; b. WFS1; and c. Sostdc1. The graph is plotted of gene expression relative to control and 7-Gy, 8-Gy, 9-Gy, and 10-Gy doses of gamma radiation. GAPDH served as internal control. To evaluate the significant fold induction as compared to the control, 1-way ANOVA and Dunnett’s multiple range test were performed (^*^P<0.05; ^**^P<0.01; ^***^P<0.001). Error bars are presented as Mean±SD.

BiP and CHOP are well-known ER chaperones and get up-regulated under conditions of ER stress. We assessed the expression of BiP, CHOP, WFS1, Sostdc1, and Nectin3 by quantitative RT-qPCR on irradiated mice hippocampus. Results demonstrate increase in CHOP and BiP gene expression with increase in dose of 7 Gy to 10 Gy and also 4 fold increase of ER stress-specific gene CHOP (***P<0.001) and 6 fold increase in BiP (**P<0.01) at 10 Gy of gamma radiation dose with respect to control (Figure 1 A–b). Furthermore, 7–10 Gy dose of gamma radiation significantly downregulates the expression of Nectin3 gene (***P<0.001), WFS1 (***P<0.001) and Sostdc1 (*** P<0.001) by 1 fold which ultimately at the 10-Gy dose (Figure 2 a–c).

### 3.2. Protein expression analysis of CHOP and ERK½

**Figure 3.**
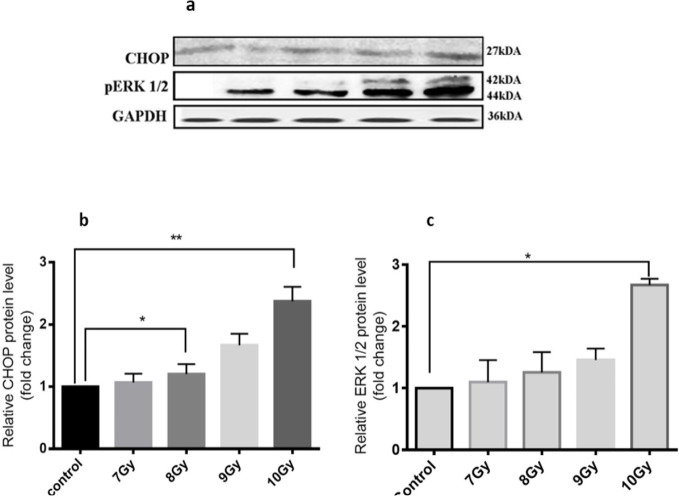
Western blot analysis of protein levels of the hippocampus after whole-body gamma radiation CHOP and ERK½ protein level were analyzed. Representative a. western blot images; b. Quantitative analysis of western blot of CHOP; and c. ERK½. Quantitative graphical representation was done using GraphPad Prism software. To represent a statistically significant protein expression as compared to the control, 1-way ANOVA and Dunnett’s multiple range test were performed with 1-way ANOVA parameters (*P<0.05;**P<0.01; ***P<0.001). Error bars are presented as Mean±SD.

CHOP gene has been commonly used as a hallmark for the ER stress-mediated apoptosis, and induction of this gene confirms ER stress-mediated apoptosis. Whole-body gamma radiation dose significantly increased (**P<0.01) expression of CHOP (Figure 3 a–b) protein level, suggesting the induction of ER-stress-mediated apoptosis in the isolated hippocampus. Significant increase in the CHOP protein expression (**P<0.01) at 10 Gy of dose gamma advocates profuse ER stress-mediated apoptosis. Also, significant (*P<0.05) increase of ERK½ protein expression at the dose range of 10 Gy dose of radiation are observed on the hippocampus (Figure 3 a, c) which further validate the involvement of ERK½ pathway, inducing ER-stress-mediated apoptosis in isolated hippocampus. Significant increase in the levels of CHOP and ERK½ suggests that IR induces hippocampal dysfunction by inducing ER-stress-mediated apoptosis, which might be further responsible for various IR-mediated hippocampal dysfunction.

## 4. Discussion

Understanding the molecular machinery responsible for radiation-induced cognitive dysfunction will provide insight into the molecular mechanism and neurobiology of stress-induced neurodegenerative disorders. Here we tried to understand how radiation onsets ER stress-induced dysfunction of the hippocampus of mice brain after whole body radiation. We observed a positive correlation with the upregulation of ER stress-specific genes and also the genes responsible for various physiological functions in the hippocampal regions. ER stress has been reported to disrupt neuronal functions and is responsible for various neurological disorders like Alzheimer disease, Parkinson disease, and Huntington disease (Siman, Flood, Thinakaran, Neumar, 2001). Radiation-induced ER stress has also been reported by us and others in various cell lines (Zhang et al., 2009).

In the present study, we found elevated expression of GRP78/BiP which has been reported earlier to have a dual role which activates under ER-stress condition as a self-defense mechanism in the cell, but under unresolved ER stress, this leads to cell death (Akutsu, Matsubara, Urashima, Komatsu, Sakata, Nishimori, et al, 2007). Another marker, CHOP, a downstream component of the ER stress pathway, is reported to get upregulated along with the induction of BiP signaling and lead the cell towards apoptotic pathway. CHOP overexpression has been linked with various neurodegenerative diseases and also targeted for development of therapeutic drugs against ER stress (Ohoka, Yoshii, Hattori, & Onozaki, Hayashi, 2005). In the present study, we observed significant upregulation with the increasing gamma radiation dose suggesting the 7 Gy gamma radiation exposure brings about significant induction of ER stress-mediated apoptosis in mice hippocampus.

Previous reports have suggested cells deficient in WFS1 are more susceptible to ER stress-mediated apoptosis. The present study we have observed a reduction of WFS1 gene with the increase of radiation dose in sync with an increase of BiP and CHOP, suggesting WFS1 plays an important role in mediating ER stress-mediated apoptosis. Also, the reduced level of WFS1 is linked with a genetic condition leading to Wolfram syndrome which causes severe depression, psychosis, or organic brain syndrome, as well as impulsive verbal and physical aggression (Takeda et al., 2001). In the current study, we observed a decrease in WFS1 gene expression with an increase of radiation dose, suggesting a link role of the WFS1 gene in inducing ER stress-mediated dysfunction in the hippocampus.

Previous reports have suggested the major role of CAM Nectin-3 in hippocampal-dependent learning and memory (Wang et al., 2013). Reduced level of nectin is associated with early life stress, disruption of synaptic contacts, and also hampered spatial memory. In our present study, we observed a dose-dependent decrease of Nectin-3 expression suggesting that 7- to 10-Gy gamma radiation have severe effect in destabilizing the hippocampal neurons, which might be disrupting hippocampal-dependent cognitive functions. Downregulation of Nectin-3 also suggests hampered episodic memories and loss of neurogenesis in the CA3 region of the hippocampus.

Sostdc1 gene, which is predominantly found in the CA2 region of the hippocampus, is also reported to play a major role in the WNT receptor signaling pathway (Inestrosa et al., 2005). We have observed significant downregulation of Sostdc1 gene on the exposure of gamma radiation, which suggests radiation causes significant changes in the WNT signaling pathway, and this might ultimately lead to various hippocampus-induced neurodegenerative diseases.

**Figure 4.**
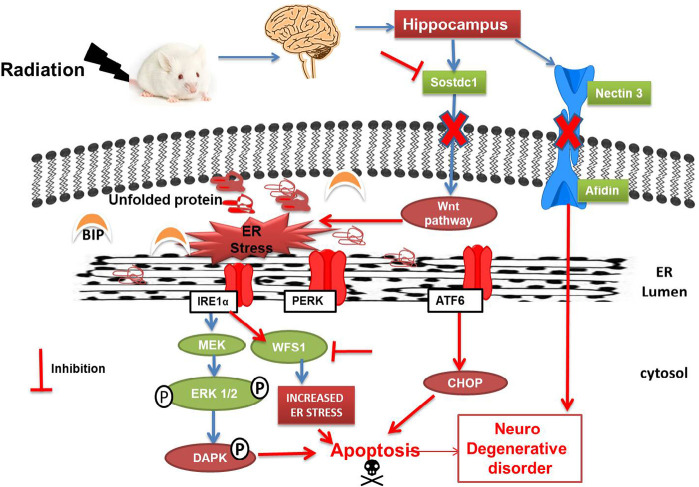
Hypothetical schematic diagram shows the mechanism of whole-body mice gamma radiation induced-ER stress-mediated disruption of hippocampal homeostasis. Radiation induces inhibition of Sostdc1 gene resulting in disruption of the WNT pathway, which might lead to ER stress of cells. Also, radiation downregulates Nectin3 resulting in hampered nectin-afadin complex and ultimately might be causing various neurodegenerative disorders. The onset of ER stress leads to the dissociation of BIP from the unfolded protein, ultimately leading to the subsequent activation of IRE1α, PERK, and AT6 pathway downstream in the ER lumen. Radiation inhibits WFS1 gene expression resulting in IRE1α-mediated apoptosis. IRE1α also activates MEK which leads to the phosphorylation of ERK½, which ultimately leads to apoptosis via phosphorylating DAPK.

Heat shock protein is said to activate various kinase pathways that control proliferation and survival like ERK½ and Akt (Mebratu & Tesfaigzi, 2009). ERK½ activation is reported to promote ER stress-induced cell death in neuroblastoma cell line (Arai et al., 2004; Mukerjee, McGinnis, Park, Gnegy, & Wang, 2000). The suppression of ERK½ or Akt activation during stress condition increases heat sensitivity. On the contrary, overexpression of wild-type ERK½ protects cells from stress (Gabai et al., 2000). In the present study, we have observed significant upregulation of ERK½ protein expression post 10-Gy exposure of gamma radiation suggesting ERK pathway mediated cell death, which might be caused via phosphorylation of pro-apoptotic signal of DAPK as also reported earlier by Mebratu & Tesfaigzi (2009) (Figure 4).

In conclusion, our present study suggests that a 7-Gy dose of whole-body gamma radiation in mice is sufficient to induce ER stress specific markers BiP and CHOP and also downregulates hippocampal genes WFS1, Sostdc1, and Nectrin3, which ultimately disrupt the hippocampal homeostasis. Significant increase of ERK½ also suggests cells innate response to overcome ER stress. As the hippocampus is involved in a wide array of physiological functions, radiation-induced damage in the hippocampus might lead to various neurodegenerative diseases such as AD, PD, etc. The present study may lead to the identification of ER stress and hippocampal genes as new markers to study radiation-induced neurodegenerative disorder induced by hippocampal dysfunction.
